# Identification of the rhizospheric microbe and metabolites that led by the continuous cropping of ramie (*Boehmeria nivea* L. Gaud)

**DOI:** 10.1038/s41598-020-77475-3

**Published:** 2020-11-23

**Authors:** Yanzhou Wang, Siyuan Zhu, Touming Liu, Bing Guo, Fu Li, Xuehua Bai

**Affiliations:** Institute of Bast Fiber Crops, Chinese Academy of Agricultural Sciences, Changsha, 410205 Hunan People’s Republic of China

**Keywords:** Ecology, Microbiology, Molecular biology, Plant sciences

## Abstract

Continuous cropping lowers the production and quality of ramie (*Boehmeria nivea* L. Gaud). This study aimed to reveal the metagenomic and metabolomic changes between the healthy- and obstacle-plant after a long period of continuous cropping. After 10 years of continuous cropping, ramie planted in some portions of the land exhibited weak growth and low yield (Obstacle-group), whereas, ramie planted in the other portion of the land grew healthy (Health-group). We collected rhizosphere soil and root samples from which measurements of soil chemical and plant physiochemical properties were taken. All samples were subjected to non-targeted gas chromatograph-mass spectrometer (GS/MS) metabolome analysis. Further, metagenomics was performed to analyze the functional genes in rhizospheric soil organisms. Based on the findings, ramie in Obstacle-group were characterized by shorter plant height, smaller stem diameter, and lower fiber production than that in Health-group. Besides, the Obstacle-group showed a lower relative abundance of *Rhizobiaceae, Lysobacter antibioticus,* and *Bradyrhizobium japonicum*, but a higher relative abundance of *Azospirillum lipoferum* and *A. brasilense* compared to the Health-group. Metabolomic analysis results implicated cysteinylglycine (Cys-Gly), uracil, malonate, and glycerol as the key differential metabolites between the Health- and Obstacle-group. Notably, this work revealed that bacteria such as *Rhizobia* potentially synthesize IAA and are likely to reduce the biotic stress of ramie. *L. antibioticus* also exerts a positive effect on plants in the fight against biotic stress and is mediated by metabolites including orthophosphate, uracil, and Cys-Gly, which may serve as markers for disease risk. These bacterial effects can play a key role in plant resistance to biotic stress via metabolic and methionine metabolism pathways.

## Introduction

Continuous cropping is defined as the agricultural practice of producing or growing a single crop on a piece of land. Long-term continuous cropping causes plant growth obstacles, mainly manifesting as decreased leaf photosynthesis and metabolism, soil-borne diseases, deteriorated soil environment, and disturbance of microbial and nematode community^1–5^, which consequently contaminate the soil, inhibits plant growth and reduces crop quality^5,6^.

Increasing evidence has revealed the changes in soil microbial diversity and structure under continuous-cropping conditions^1,7,8^. Continuous cropping usually lowers the abundance of beneficial microbes, such as *Azotobacter*, *Rhizobium,* and *Nitrobacteria*, while similarly enhancing the growth of harmful microbe in soils^9–12^. For instance, Tan et al. in their study reported that the continuous cropping of *Panax notoginseng* reduced the fungal diversity in the rhizospheric soil and roots^3^. Also, Han et al. that continuous cotton cropping for 5 years not only reduced the diversity of nutrition related bacteria but also lowered the relative abundance of ammonifying bacteria, nitrifying bacteria, and aerobic nitrogen-fixing bacteria. Changes in the diversity of soil bacteria are correlated with soil physicochemical properties, for example, pH value, moisture, salinity, porosity, available nitrogen, and organic matter^13–15^. Accordingly, continuous cropping practices lower crop yields and quality by affecting the biosynthesis and metabolism of active plant components^16^.

Ramie (*Boehmeria nivea* L. Gaud), also known as “China grass,” is a perennial, diploid (2n = 28) and herbaceous plant of the *Urticaceae* family. Ramie is widely cultivated owing to its bast fiber, which offers excellent properties such as excellent thermal conductivity and high tensile strength^17^. Ramie fiber is extracted from stem bast and poses numerous excellent characteristics, for example, long strands, smooth texture, and high tensile strength^18^. Previous studies reported that continuous cropping of ramie reduces plant height, stem diameter, and fiber yield^5,12,19^. In our previous studies, we showed that the stem growth of ramie is impeded by continuous cropping^12^. Moreover, we found that the abundance of *Firmicutes* was correlated positively with the plant height, stem diameter, and ramie fiber yield. However, as the duration of continuous planting increases, fiber production decreases^12^. The above studies revealed the obstacles in the continuous cropping of ramie. However, none of the reports indicated whether soil microbial communities and metabolites were associated with continuous cropping obstacles.

In production practice, we found an interesting phenomenon: After 10 years of continuous cropping (single variety), ramie planted in some portions of the land were characterized by weak growth and low yield (Obstacle-group), on the other hand, ramie planted in the other portions exhibited healthy growth (Health-group). Thus, we hypothesized that this difference may be attributed to the effects of soil microorganisms and could be revealed by soil metagenomics and metabolome analysis. This present study attempted to provide a better understanding of the relationship between soil bacteria and metabolites and continuous cropping obstacles and to elucidate on the growth of ramie under continuous cropping practice.

## Results

### Continuous cropping of ramie

**Figure 1:**
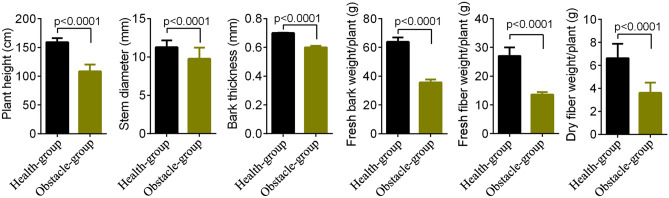
Changes in ramie growth and fiber traits through continuous cropping based on data collected from 30 individual plants in each group. Differences are analyzed using a t-test.

After 10 years of continuous cropping (single variety), ramie planted in some portions of the grew healthy (defined as Health-group) whereas, ramie planted in the other portions grew weak in and with low yields (defined as Obstacle-group). Soil samples in Obstacle-group had lower contents of available P and K, and higher content of total K than Health-group (p < 0.05, Table 1). The values of plant height, stem diameter, bark thickness, weight, and fresh and dry fiber weight in the Obstacle-group were lower compared to those in Health-group (p < 0.0001, Fig. 1). These findings confirmed that growing the same ramie variety in different continuous cropping lands greatly varies.

**Table 1:** Differences in soil chemical parameters between Health-group and Obstacle-group.

Parameters	Health-group	Obstacle-group	p
Total N (%)	1.35 ± 0.03	1.30 ± 0.05	0.152
Total P (%)	0.08 ± 0.00	0.07 ± 0.01	0.104
Total K (%)	3.42 ± 0.03	3.50 ± 0.02	0.018
Available P (mg/kg)	29.03 ± 0.15	20.27 ± 0.15	< 0.0001
Available K (mg/kg)	101.33 ± 2.08	86.67 ± 1.53	0.0006

Differences are analyzed using a t-test.

### Illumina gene sequencing

**Figure 2:**
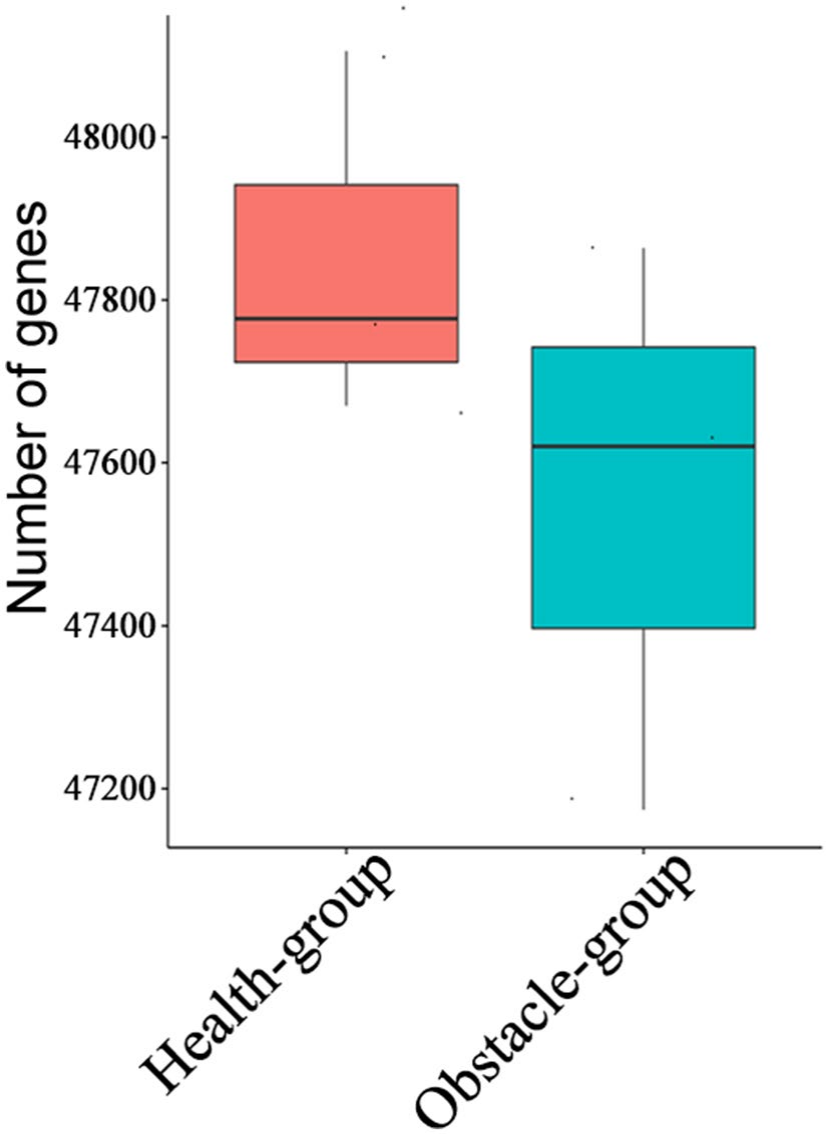
Differences in microbial gene number and kingdom abundance between the two groups. Boxplots indicate the minimum, maximum, median and upper and lower quartiles of the number of genes in each group. In a boxplot, the black line indicates the median.

Illumina sequencing generated a total of 326.58 M clean reads from 6 rhizosphere soil samples with an average Q30 value of 94.59% and GC content of 64.37%. A total of 10,450,4244 scaffolds and 80,931 open reading frames (ORFs, longer than 200 bp) were retrieved from the data (Table S1). Additionally, 435,887 non-redundant gene catalogs or clusters were identified, including 49,444 annotated genes to bacterial taxonomy. Expression analysis showed that 5517 clusters were differentially expressed between the two groups. Notably, the numbers of annotated genes in the two groups were different (Fig. 2). About 98.26% of the clean reads originated from bacteria, followed by 1.28% and 0.3% from archaea and virus, respectively.

### Identification of different microbe between the two groups

**Figure 3:**
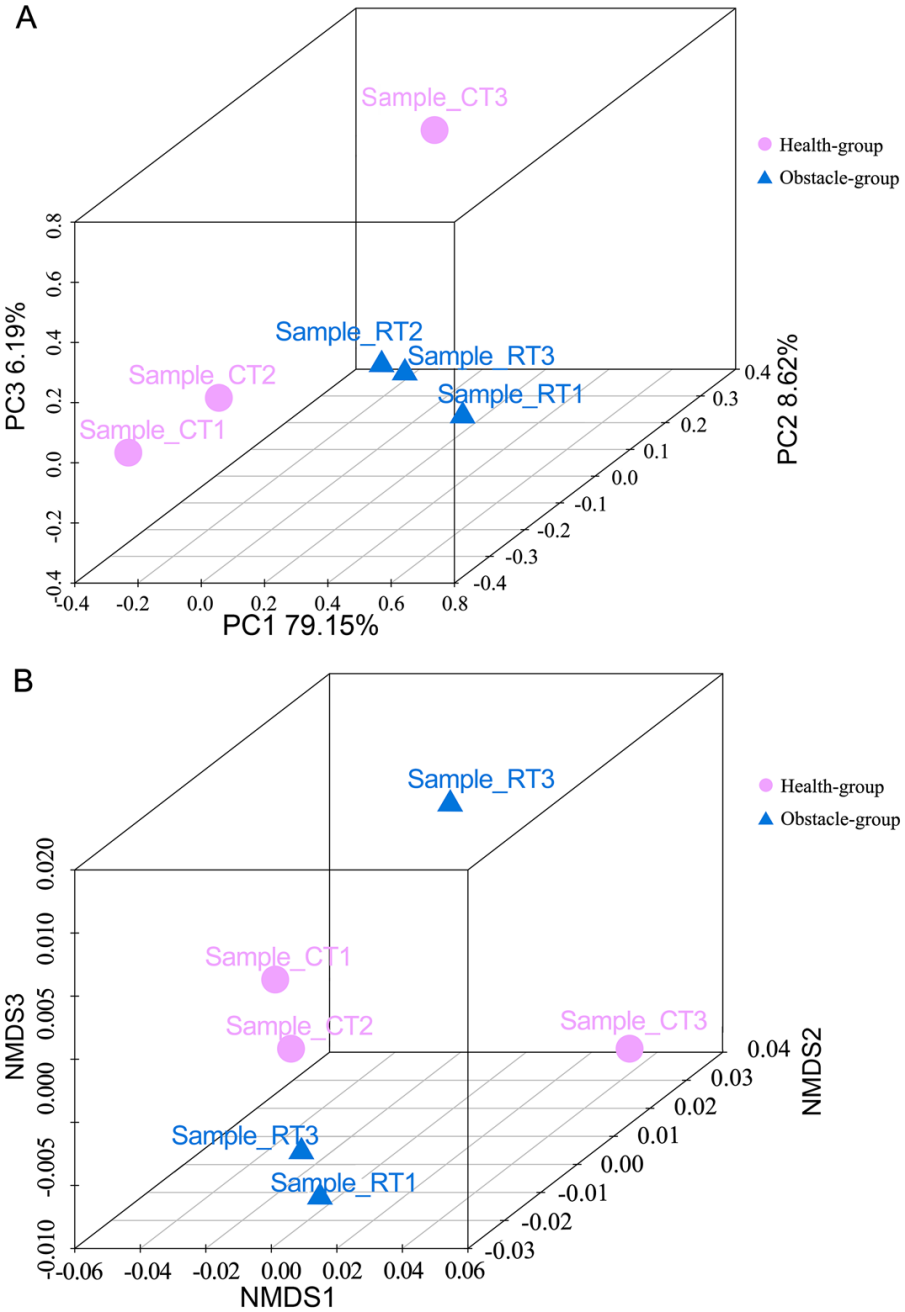
Beta diversity among samples sequenced. (**A**) The principal co-ordinates analysis (PCoA) results. (**B**) The Nonmetric Multidimensional Scaling (NMDS) analysis results. Both analyses were performed based on the Euclidean distance algorithm.

**Figure 4:**
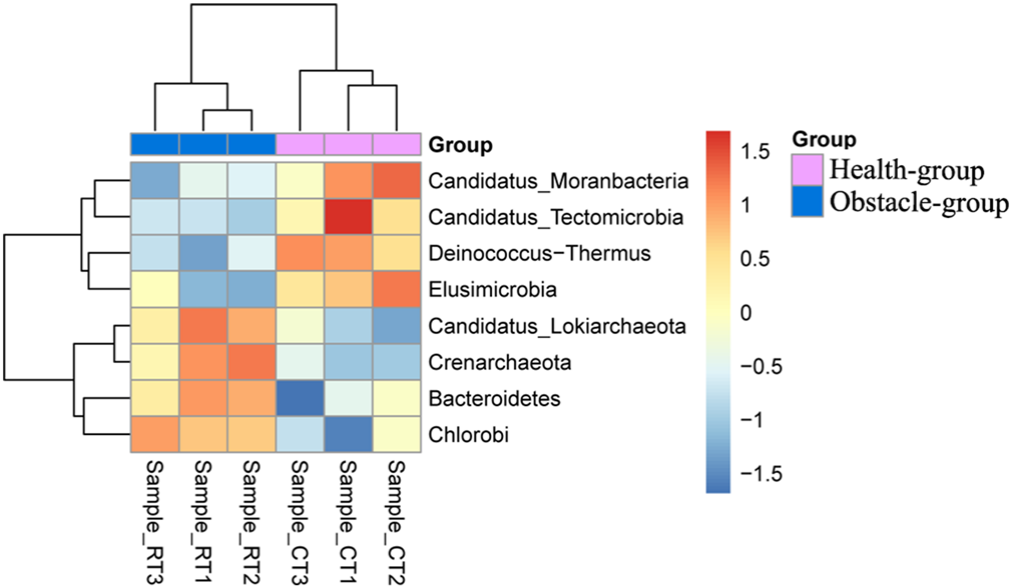
A heatmap of the 8 phyla among samples. The dendrogram to the left and top of the heatmap shows the clustering tree. The box was colored based on the standardized relative abundances data. The redder color shows a higher abundance of the phyla, and the blue color shows a lower abundance.

Principal coordinates analysis (PCoA) and Nonmetric Multidimensional Scaling (NMDS) analyses demonstrated different beta diversity among all samples (Fig. 3A,B). Samples in Health-group were clustered together in both PCoA (PC1 = 79.15%, PC2 = 8.62%, and PC3 = 6.19%) and NMDS. Similarly, samples in Obstacle-group were clustered together. Among the top 10 taxonomies (from species to phylum), no significant difference was found in the abundance of microbes. Then, we further analyzed the microbes with a lower relative abundance. We found that the relative abundances of 8 phyla (Fig. 4, two *Archaea* phyla), 41 families (Fig. S1), 132 genera and 493 species (Table S2) were different between Health- and Obstacle-group. For instance, the relative abundance of *Acidithiobacillaceae* (0.023 ± 0.002%, *Proteobacteria*), *Ectothiorhodospiraceae* (0.520 ± 0.016%, *Proteobacteria*) and *Xanthomonadaceae* (0.973 ± 0.020%, *Proteobacteria*) family in Obstacle-group was higher than that in Health-group (0.019 ± 0.000%, 0.480 ± 0.055%, and 0.921 ± 0.023% correspondingly; p < 0.05, Fig. S1). The abundance of *Rhizobia* (*Rhizobiaceae*) was lower in Obstacle-group than in Health-group (1.073 ± 0.014% vs. 1.120 ± 0.008%, p < 0.05). Besides, the abundance of two virus families *Podoviridae* and *Myoviridae* was higher and lower in Obstacle-group compared with Health-group, respectively (*Podoviridae* 0.030 ± 0.003% vs. 0.038 ± 0.003%; *Myoviridae* 0.018 ± 0.001% vs. 0.015 ± 0.000%, p < 0.001). At the species level, the relative abundance of *Janthinobacterium lividum* (0.019 ± 0.001% vs. 0.014 ± 0.001%, p < 0.05), *Bradyrhizobium japonicum* (0.119 ± 0.002% vs. 0.102 ± 0.002%, p < 0.05) and *Lysobacter antibioticus* (0.030 ± 0.002% vs. 0.023 ± 0.001%, p < 0.05; Table S2) were higher in Obstacle-group compared to Health-group (p < 0.05). These findings demonstrated that the growth of plants under continuous-cropping conditions is correlated with the changing diversity of soil microbe, especially the bacteria.

### GC–MS/MS metabolomics

TICs (total ion currents) revealed 554 peaks detected from all samples (including quality control samples). Metabolite annotation results showed 190 identified metabolites. PCA (Fig. S2A) and OPLS-DA (Fig. S2B) results showed that there were different metabolites in soil and root between the two groups. Moreover, we identified 20 and 31 differential metabolites in the soil and root samples between the two groups, respectively (Table S3), including 3 overlapping metabolites (threitol, glycerol, and 2-Deoxyerythritol).

### Identification of the differential metabolites in soil samples

**Figure 5:**
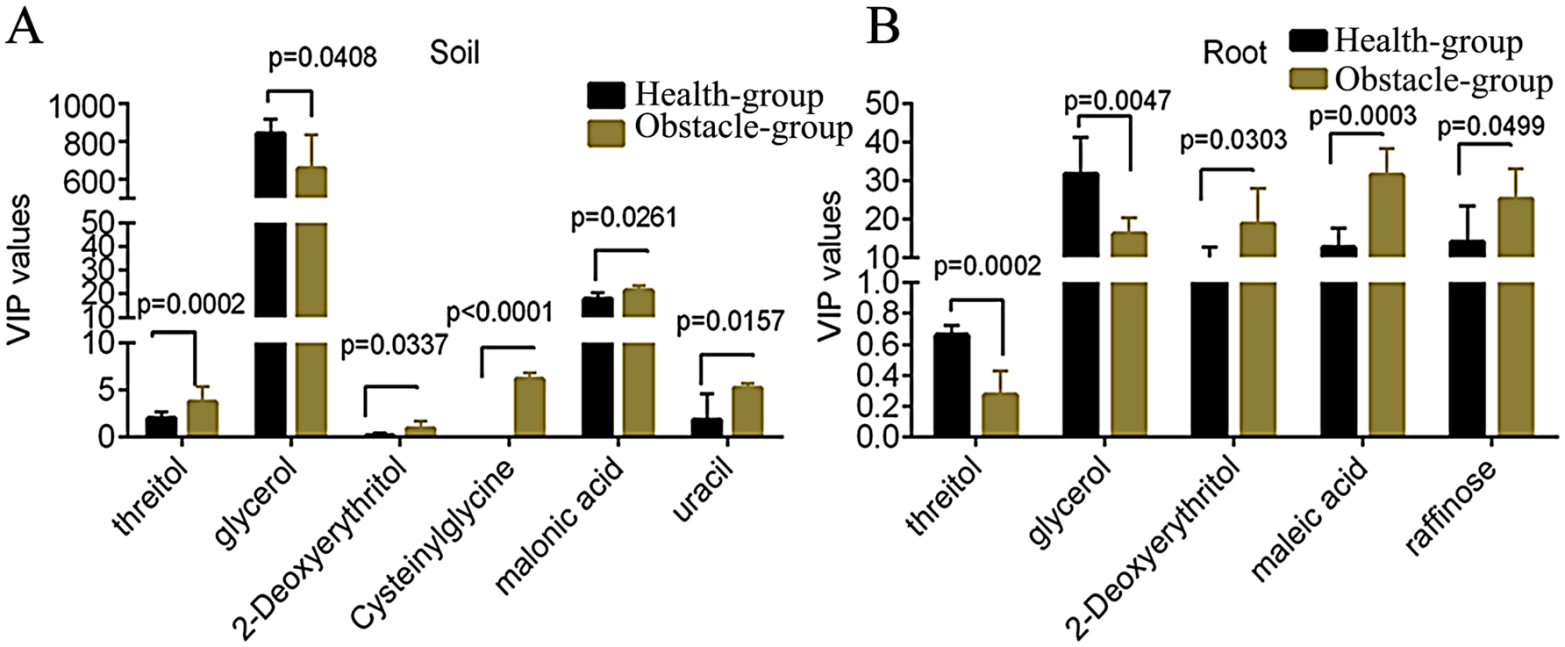
Different metabolites from the soil and root samples between the two groups. Several differential metabolites in soil (**A**) and root (**B**) samples between the two groups, respectively. VIP, variable importance in projection. VIP scores estimate the importance of each variable in the projection used in a PLS model. The p-value is analyzed using a t-test. N/B: the Y axes have been divided into two or three segments for a clearer representation of smaller bars. P values are marked in the figure (n = 6).

From the soil samples, there were higher levels of threitol, 2-Deoxyerythritol, uracil, malonic acid, and Cys-Gly (Cysteinylglycine and lower glycerol level in Obstacle-group than in Health-group (Table S3, Fig. 5A). KEGG (Kyoto Encyclopedia of Genes and Genomes) pathway enrichment analysis showed that these 20 different metabolites in soil samples were enriched in 17 pathways. Notably, Cys-Gly, orthophosphate, uracil, malonate, and glycerol had a higher frequency in these pathways. Moreover, Uracil was involved in multiple pathways, such as metabolic pathway, pantothenate and CoA biosynthesis, pyrimidine metabolism, among others, whereas malonate was associated with pyrimidine metabolism and beta-Alanine metabolism.

### Identification of the differential metabolites in root samples

From the root samples, the level of glycerol and threitol was higher in Obstacle-group compared to Health-group (Fig. 5B). On the contrary, the level of 2-Deoxyerythritol, maleic acid, and raffinose in the Obstacle-group was higher than in Health-group (p < 0.05). The KEGG enrichment analysis showed that 90 pathways were enriched, including Carbon metabolism (e.g. fumarate, 3-Hydroxypropanoate, citrate, d-Gluconic acid, d-Glucono-1,5-lactone, and pyruvate), Metabolic pathways (e.g. fumarate, 3-Hydroxypropanoate, citrate, glycerol, myo-Inositol, alpha,alpha-Trehalose, d-Gluconic acid, cysteamine, pyruvate, and Phytosphingosine), Biosynthesis of phenylpropanoids, and Biosynthesis of plant secondary metabolites (e.g. fumarate, citrate, gallate, l-Phenylalanine and pyruvate; Table S4). Of all the enriched differential metabolites in the pathways, orthophosphate, uracil and Cys-Gly were found to exhibit a high frequency.

### Integrated analysis of the metagenome and metabolomics

**Figure 6:**
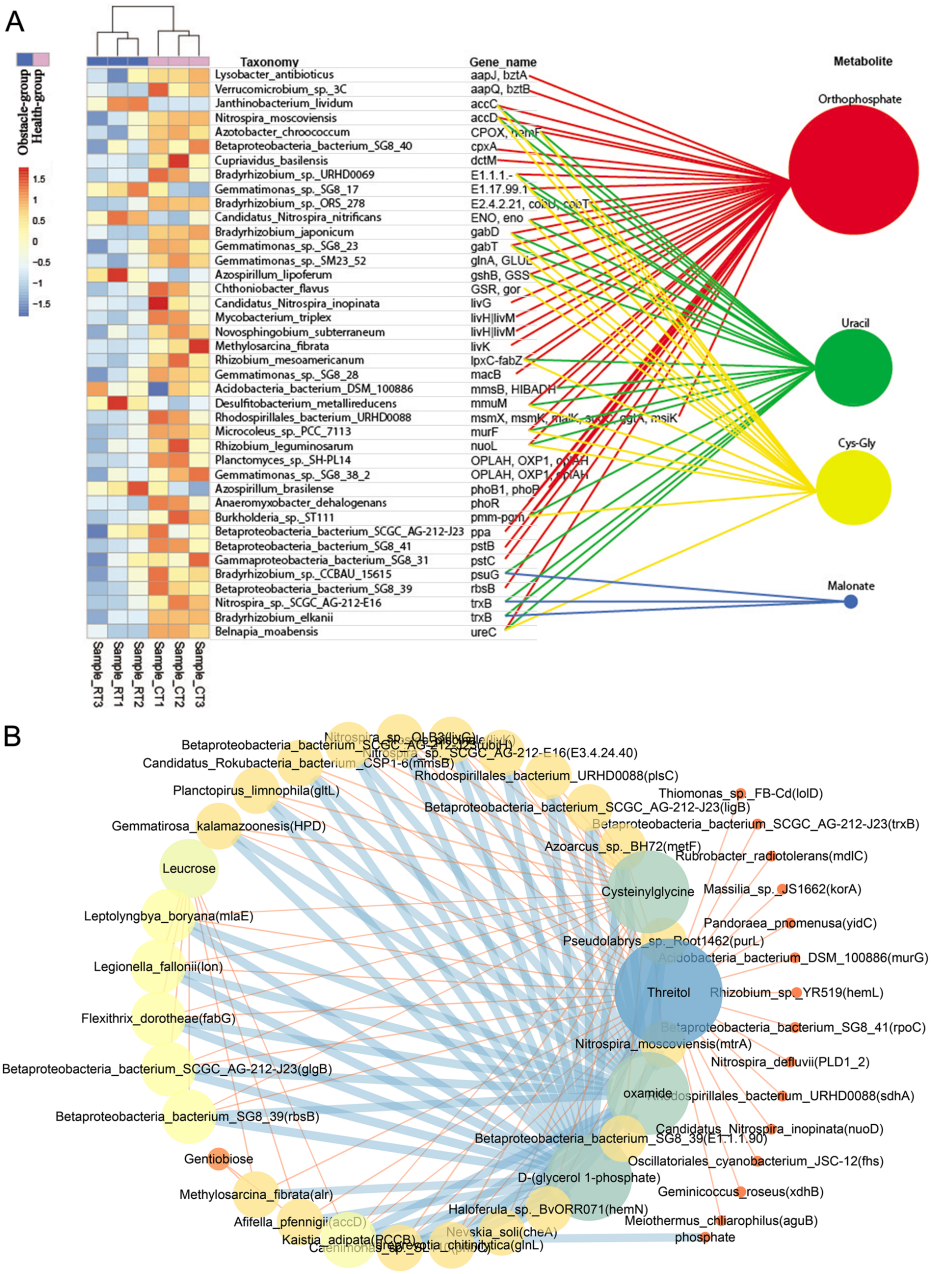
The integrated analysis of different bacteria, metabolites and bacterial genes. (**A**) The relationship between different metabolites and different genes enriched in the same pathway. The heatmap shows the relative abundance of bacteria in different groups, the redder the color, the higher the abundance. The colored scale shows the level of abundance based on the standardized relative abundances data. Gene name and bacterial name horizontally correspond to each other, the larger the size, the higher the interaction. (**B**) The interacting network among the top 50 metabolites and genes, the darker and bigger the node, the richer the interaction with the genes.

To identify the correlation between the differential microbial genes and metabolites in the two groups, we identified the same pathways associated with the differential genes and metabolites in soil and root samples. Of note, the oxidative phosphorylation (ko00190), pyrimidine metabolism (ko00240), beta-alanine metabolism (ko00410), glutathione metabolism (ko00480), metabolic pathways (ko01100), ABC transporters (ko02010), and two-component system (ko02020) were found to be correlated with the content of metabolites (e.g., orthophosphate, uracil, malonate and Cys-Gly) through the genes expressed in 5 *Archaea*, one virus and 206 bacteria (Table S3). For instance, *Archaea* N*itrososphaera viennensis*, Candidatus *Nitrososphaera gargensis* and Candidatus *Nitrososphaera evergladensis* regulated two-component system (ko02020) and metabolic pathways (ko01100) by changing the metabolomic profiles of orthophosphate, uracil, and Cys-Gly. Genes expressed in *Archaea,* for example, cmpC glutamate dehydrogenase (GDH2) and glrR are listed in Table S3. Of which, *WspR*, expressed in *Azoarcus* sp. PA01 and *Planococcus* sp. PAMC_21323, regulated orthophosphate metabolism and was involved in the two-component system pathway; trxB was expressed by *Nitrospira sp.* SCGC_AG-212-E16 and *Bradyrhizobium elkanii*, which regulated pyrimidine metabolism by regulating the metabolism of uracil and malonate. The f accD (*Nitrospira moscoviensis*) and accC (*Janthinobacterium lividum*) genes could regulate orthophosphate, uracil, and Cys-Gly and were enriched in metabolic pathway (ko01100) (Table S3). Besides, we identified several genes involved in methionine metabolism, which regulates orthophosphate, uracil and Cys-Gly metabolism, they include gshB (encodes glutathione synthase, GSH) in *Azospirillum lipoferum*; pepA (encodes leucyl aminopeptidase, LAP) in *Elusimicrobium minutum*; phoB1 (encodes alkaline phosphatase synthesis response regulator PhoP) in *A. brasilense*; and mmuM (encodes homocysteine S-methyltransferase, HMT) in *Desulfitobacterium metallireducens* (Fig. 6A). In addition, an interaction network was constructed to reveal the relationship between metabolites and candidate genes (Fig. 6B). Results showed that Threitol, Cys-Gly, oxamide and d-(glycerol 1-phosphate) were highly associated with the genes. These findings uncovered the crucial roles of soil bacterial diversity in the rhizospheric and root metabolites, in particular, Cys-Gly, which participated in multiple pathways and showed rich interactions with other compositions.

## Discussion

Continuous cropping obstacles are primarily related to disorders or deterioration of rhizosphere microorganisms^20,21^. Our present study confirmed that continuous cropping of ramie is correlated with the changing rhizospheric soil microbes and soil metabolomics as well as the root metabolism. The present study identified that the abundance of some bacteria, including *Rhizobia, L. antibioticus,* and *Bradyrhizobium japonicum*, were lower in Obstacle-group compared to Health-group, a finding that concurs with previous reports^22–25^. A higher abundance of *Rhizobia* can increase soil biomass, as was confirmed in previous experiments^26–29^. Among many soil bacteria, *Rhizobia* can produce higher levels of indolacetic acid (IAA)^30^, which is one of the most physiologically active auxins. IAA is a common product of l-tryptophan metabolism produced by several microorganisms including Plant Growth Promoting Rhizobacteria (PGPR)^31^. The endogenous or exogenous IAA can stimulate cell elongation by modifying certain conditions, for example, increasing in osmotic contents of the cell, increasing the permeability of water into cells, reducing wall pressure, increasing cell wall synthesis and inducing specific RNA and protein synthesis^32^. In addition to *Rhizobia,* we identified two bacteria (including *A. lipoferum* and *A. brasilense*) that were highly abundant in the Obstacle-group compared to Health-group. Notably, *A. lipoferum* and *A. brasilense* are growth-promoting bacteria and are capable of impoving plant resistance to abiotic stresses partially via enhancing plant abscisic acid level^33,34^. Both *A. lipoferum* and *A. brasilense* can also produce IAA^35^. For instance, Mazhar et al. showed that the inoculation of *A. lipoferum* ameliorated salinity-induced growth stress in wheat by promoting production of plant osmolytes (proline and soluble sugars). However, a recent study performed by Rivera et al. demonstrated that L-amino acids (e.g., L-Leu, L-Phe, L-Ala, L-Ile, and L-Pro) inhibited the growth of bacteria and biosynthesis of IAA. Elsewhere, *A. brasilense* inoculation could increase the uptake of nitrate, a substance of GSH^36^, whereas it elevated nitrogenase release, thereby revealing superior plant growth promotion^37^. Moreover, the overexpression of GSH in plants has been shown to contribute to improved plant stress tolerance and productivity^38^. It is worth noting that *A. lipoferum* expresses the GSH encoding gene gshB, while *D. metallireducens* expresses a gene mmuM encoding HMT involving in plant tolerance and defense response^39,40^. Although no l-amino acids were detected among the differential metabolites in soil and ramie root, our study uncovered that these bacterial genes regulated the metabolism of orthophosphate, uracil, and Cys-Gly, thus participates in metabolic pathway and methionine metabolism. Therefore, we speculated that the increased abundance of *A. lipoferum* and *A. brasilense* in the soil may be associated with the resistance to the continuous cropping induced abiotic stress in ramie root. In the present study, *L. antibioticus* was revealed to play important roles in various metabolic pathways via the regulation of metabolites such as orthophosphate, uracil, and Cys-Gly. Moreover, *L. antibioticus* could produce phenazine antibiotics^41^. In the recent past, Laborda et al. showed that *L. antibioticus* OH13 produced antifungal p-aminobenzoic acid. Another study reported that *L. antibioticus* exhibited biocontrol potential as it produced 4-hydroxyphenylacetic acid and several lytic enzymes against phytophthora blight^42^. In summary, these studies showed that *L. antibioticus* exerts a positive effect on plants in fighting biological stress. Low levels of *L. antibioticus* in the Obstacle-group may imply a decline in plant defense mechanism. Moreover, metabolites such as orthophosphate, uracil, and Cys-Gly may serve as mediators for *L. antibioticus* to play an active role. In addition, *Bradyrhizobium japonicum,* a microsymbiotic nitrogen-fixing bacteria, is known to enhance legume-root nodulation, which when introduced when planting, improve crop yields, especially legumes^43^. As far as we know, no report has explored whether *Bradyrhizobium japonicum* is related to the growth of ramie. However, based onour findings, we speculate that *Bradyrhizobium japonicum* may also play an active role in maintaining the health of ramie.

Our metagenome analysis showed a lower relative abundance of several soil probiotics in the Obstacle-group, which may have been induced by long-term continuous cropping. Additionally, the NMDS and PCoA analyses using different methods revealed the distinct separations among samples from different groups. This showed that differences exist in the microenvironment of rhizosphere soil between the Obstacle-group and Health-group. The PCoA based on the weighted UniFrac algorithm clearly demonstrated the variations among different groups. And in the dimension of PC1, the two groups can be distinguished to the greatest extent (79.15%). These differences in soil microorganisms might be the most important factor that ultimately led to a greater difference between the two groups. Han et al. showed that 5-year continuous cropping of cotton lowered not only the richness of nutrition bacteria type in soil but also the abundance of ammonifying bacteria, nitrobacteria, and aerobic nitrogen-fixing bacteria. Also, a different study reported that two plant growth-promoting bacteria *Azospirillum brasilense* Cd and *B. pumilus* ES4 could enhance the growth of microalga by inducing the production of total carbohydrates, chlorophyll a and total lipids in microalga^34^. Besides, numerous previous studies found that *Bradyrhizobium* and *Rhizobium* (*Proteobacteria*) are symbiotic bacterial partners that promote the formation of nitrogen-fixing nodules on legumes, thereby enhancing the plant growth, resistance, and tolerance to abiotic stresses and pathogens^44–46^. Similarly, our present study reported higher relative abundances of *R. leguminosarum*, *A. chroococcum* and *R. mesoamericanum* (*Proteobacteria*) in the Health-group than that in the Obstacle-group, which was inversely associated with the inhibition of ramie growth. The above-mentioned bacteria potentially play a key role in keeping plants healthy. Previous findings revealed the *Lysobacter* species as a potential source of novel antimicrobial plant metabolites^41,47,48^. Notably, *L. antibioticus* has been revealed to be capable of producing phenazine antibiotics^41^. For example, a recent study by Laborda et al. showed that *L. antibioticus* OH13 produced antifungal p-aminobenzoic acid. In our study, we suggested that the lower level of *L. antibioticus* in the Obstacle-group may signify a decline in plant defense.

## Conclusions

Soil bacteria may primarily affect health and obstacles to the growth ramie after long-term continuous cropping. Bacteria such as *Rhizobia* can synthesize IAA and are likely to reduce the biotic stress of ramie. For instance, *L. antibioticus* exerts a positive effect on plants in fighting biotic stress. In this study, we showed that lower levels of *L. antibioticus* in the Obstacle-group may imply a decline in plant defense. Metabolites including orthophosphate, uracil, and Cys-Gly might serve as mediators for *L. antibioticus* to play an active role. Notably, subjecting ramie to biological stress may increase in abundance of *A. lipoferum and A. brasilense*. Consequently, the bacterial changes are associated with various metabolic pathways through the regulation of metabolites such as orthophosphate, uracil and Cys-Gly. Particularly, Cys-Gly participated in multiple pathways and showed rich interaction in the interaction analysis. A low abundance of beneficial bacteria in Obstacle-group might be associated with the resistance to the continuous cropping induced abiotic stress in ramie root. These bacterial changes may play a key role in plant resistance to biotic stress via the metabolic and methionine metabolism pathways. Thus, this present study provides novel insights into the association of soil microbe and metabolome. These findings might show some indications on the agricultural practice for continuous ramie cropping.

## Methods

### Plant materials and sample collection

The ramie cultivar (*Zhongzhu NO.1*) was planted at the experimental field in the Institute of Bast Fiber Crops, Chinese Academy of Agricultural Sciences, Changsha, China (N 28° 12′ 35.91″, E 112° 41′ 51.07″). The seeds of *Zhongzhu NO.1* were obtained from the Seed resource bank of the Institute of Bast Fiber Crops. The experiment commenced in March 2008 and proceeded up to June 2017, during which a single-variety *Zhongzhu NO.1* was grown through a continuous cropping system (3 seasons per year, 10 consecutive years). The test field was 15 acres (areas were close to each other with a similar initial soil structure). All the acres were farmed under the same cropping system and conditions, including annual planting time, harvest time, fertilization and irrigation measures, etc. Routine agronomic management and fertilization were performed annually during the ramie growing period. In the later period of the experiment, we found that the ramie planted in some portions of the land grew well (after 10 years of continuous cropping, some ramie were healthy, and were defined as Health-group), while the ramie planted the other portions had obvious growth obstacles due to continuous cropping (mainly characterized by short plants and low fiber production, and were defined as Obstacle-group). Rhizosphere soil samples were collected from the Health-group and Obstacle-group whereby a total of 30 soil samples were obtained in each group on June 23, 2017. The fields in the two groups were about 10 m apart, and each field was separated by a 1.5-m-wide ditch. Every 5 samples were pooled, and a total of 6 rhizosphere soil samples were prepared. The pooled soil samples in each group were divided into 2 portions, for metagenomics sequencing analysis (randomly selecting three samples, n = 3) and metabolome analysis, respectively. We also collected the ramie root samples. A total of 30 root samples were collected, every 5 samples were pooled, and a total of 6 ramie root samples were prepared. All the 6 root samples were used for metabolome analysis. All collected samples were stored at − 80 ℃ immediately after preparation.

### Detection of soil chemical properties

The soil chemical properties, including the contents of available nitrogen (N), phosphorus (P), potassium (K), and total N, P, K were measured according to the methods reported previously^49^. In each group, 15 samples were randomly collected from 0 to 60 cm soil, mixed into three pools, air dried and ground. Soil total N, P and K was detected using the GB 7173-1987 (NY/T 53-1987, semi-micro Kjeldahl method), GB 9837-1988 (NaOH melt-colorimetry method with molybdenum and antimony) and GB 9836-1988 (NY/T 87-1988) national standard of China, respectively. Soil available P and K content was measured using the Bray and Kurtz 1 extraction solution (0.025 M HCl in 0.03 M NH4F) and colorimetry (660 nm), DB13/T 844–2007 (CH_3_COONH_4_ extraction-flame photometric method), respectively. Each determination was repeated at least three times.

### Determination of the plant growth and ramie quality indexes

For the determination of ramie growth and quality, the plant height (cm), stem diameter (mm), bark thickness (mm), fresh bark weight (g), fresh and dry fiber weight (g) were measured and recorded according to the previous report^50^.

### DNA extraction, library preparation, and Illumina sequencing

Rhizospheric soil samples (n = 6) were collected from the two groups in May 2017. Soil samples were subjected to DNA extraction using a MoBio PowerSoil DNA Isolation Kit (MoBio Laboratories, Carlsbad, CA, USA) following the manufacturer’s instructions. DNA concentration, purity and integrity were determined using NanoDrop ND-1000 spectrophotometer (NanoDrop Technologies, Wilmington, DE, USA) and 1% agarose gel electrophoresis. DNA samples were fragmented, ligated to adapters and enriched standardly. TruSeq DNA PCR-Free Sample Preparation Kit (Illumina, San Diego, CA, USA) was used for the construction of DNA libraries, which were then evaluated using Qubit 2.0 Fluorometer (Thermo Scientific, MA, USA) and Agilent Bioanalyzer 2100 system (Agilent Technologies, Santa Clara, CA, USA). Illumina HiSeq2500 platform (250 bp paired-end reads) was employed for the sequencing.

### Sequencing data preprocessing

The paired-end raw reads in the format of FASTQ were quality controlled using Trimmomatic by trimming and filtering adaptors and low quality reads. SOAP denovo (v4.5.4; https://soap.genomics.org.cn/) was used for the metagenome assembly^51^. ORF prediction of assembled scaffolds was performed using prodigal (v2.6.3) and was translated into amino acid sequences. All effective tags were clustered by CDHIT (v4.5.4; https://www.bioinformatics.org/cd-hit/)^52^. With the thresholds of 95% identity and 90% coverage, a non-redundant gene set was assembled. The relative abundance of each gene in the clean reads was analyzed using fast gapped-read alignment with Bowtie2 (v2.1.0)^53^ by blasting to the non-redundant gene catalog. GeneMark.hmm (v3.26) was used to predict the potential protein-coding region in a DNA sequence. Blastp (v2.2.28 + , https://blast.ncbi.nlm.nih.gov/Blast.cgi) was employed for the alignment in NCBI nonredundant (NR) database (e-value = 1e−5). Then the sum of the gene abundance was calculated and the abundance of microbe at different taxonomies was obtained accordingly. Heatmap of the abundance profile was constructed using R (v3.2.0). The annotation of the gene products were performed in databases including NR, SWISSPROT, COG (Clusters of Orthologous Groups of proteins), Gene Ontology (GO) and KEGG with the threshold of e ≤ 1e−5. PCoA and NMDS analysis was performed based on the Euclidean distance algorithm to identify the beta diversity among samples and groups.

The differentially expressed genes in metagenome between the two groups were identified using ANOVA in QIIME (V1.8, https://qiime.org/scripts/alpha_rarefaction.html). The KEGG pathways associated with differentially expressed genes were identified in KEGG database (https://www.genome.jp/KEGG/pathway.html) with the threshold of p < 0.05.

### Sample preparation for metabolome analysis

A total of 60 mg ramie root samples were collected from ramie plants monocultured continuously for 10 years in soil without (Health-group, n = 6) and with (Obstacle-group, n = 6) continuous cropping, so did as the rhizosphere soil samples (n = 12, 1 g). 60 mg of root samples were placed into 1.5 mL EP tube, incubated with 360 μL pre-cooled methyl alcohol and 40 μL l-2-chloro-phenylalanine (Shanghai hengchuang biotechnology co., LTD., Shanghai, China) at 80 °C for 2 min, followed by grinding into homogenate using an automated grinder (JXFSTPRP-24/32, Shanghai Netcom Industries Development Co., Ltd. Shanghai, China; at 60 Hz for 2 min) and ultrasonic cleaner (SB-5200DT, Ningbo Scientz Biotechnology Co., Ltd, Ningbo, China; for 30 min). Samples were then incubated with 200 μL chloroform with sonication for 30 min, centrifuged at 12,000 rpm, 4℃ for 10 min, and dried in a vacuum centrifuge concentrator (LNG-T98, Huamei Biochemistry Instrument, Taicang, China). For soil samples, 1 mL 50% pre-cooled methyl alcohol and 20 μL l-2-chloro-phenylalanine were used for sample extraction, followed by setting at − 20 °C for 2 min and grind (at 60 Hz for 2 min). Homogenates were centrifuged and supernatants were collected, dried, dissolved into 50% pre-cooled methyl alcohol, vortex oscillated for 60 s, ultrasonic-treated for 30 s, and finally centrifuged (at 12,000 rpm, 4℃ for 10 min) and dried. All samples were subjected to treatments with methoxamine hydrochloride pyridine under rotary (2 min) and concussion (at 37 °C for 90 min); and further incubation with BSTFA (containing 1% TMCS) and 20 μL n-hexane by rotary (2 min) and concussion (at 70 °C for 60 min).

### Preparation of quality control (QC) samples

QC samples were prepared for the non-targeted metabolomics analysis by pooling equal aliquots of individual sample (n = 6) within each group. Three QC samples were prepared.

### Non-targeted chromatographic parameters

The analytical instrument used in this experiment is a 7890b-5977a gas chromatograph-mass spectrometer (GS/MS) from Agilent Technologies Inc. (USA). The chromatographic conditions are: DB-5MS column (30 m × 0.25 mm × 0.25 μm; Agilent J&W Scientific, Folsom, CA, USA); high purity helium (99.99%) with a flow rate of 1.0 mL/min; injection temperature of 260 °C. The temperature of column was increased from 60 to 125 °C at a speed of 8 °C/min, then increased to 210 °C at a speed of 4 °C/min, to 270 °C at a speed of 5 °C/min, to 305 °C at 10 °C/min and kept for 3 min. MS conditions are: electron impact ion (EI) source at 230 °C; quadrupole temperature at 150 °C; 70 eV high-energy. EI-MS spectra were recorded at a scan range of m/z 50–500. System stability and accuracy was validated using QC samples with an interval of 5 samples.

### Non-targeted metabolomics data analysis

MS raw data (total ion current, TIC) was converted into file format using ChemStation (version E.02.02.1431, Agilent Technologies Inc). ChromaTOF (version 4.34, LECO, St Joseph, MI) was used to analyze the data, and NIST and Fiehn database were used for the annotation of the metabolites. After alignment with Statistic Compare component, the ‘raw data array’ (.cvs) was obtained from raw data including peak names, retention time-m/z and peak intensities. All internal standards and pseudo positive peaks were removed. Data was transformed by log2 and then imported into SIMCA software package (14.0, Umetrics, Umeå, Sweden). Unweighted principle component analysis (PCA) and (orthogonal) partial least-squares-discriminant analysis (OPLS-DA, with sevenfold cross validation and response permutation testing, 200 times randomly permutated) were performed to visualize the metabolism difference between groups. Metabolites with variable important in projection (VIP) > 1 and p value < 0.05 by two-tailed Student’s t-test were used for identification of differential metabolites. Metabolites between groups with |fold change (FC)|≥ 1 were considered as differential metabolites. The KEGG pathways associated with the differential metabolites were identified from KEGG database (https://www.genome.jp/KEGG/pathway.html) with the threshold of p < 0.05.

### Statistical analysis

GraphPad Prism 6 software was used for data statistical analysis. Data were expressed as the mean ± SD and differences between groups were analyzed by t-test. A value of *p* < 0.05 was considered statistically significant.

## Supplementary information

Supplementary Legends.

Supplementary Figure S1.

Supplementary Figure S2.

Supplementary Table S1.

Supplementary Table S2.

Supplementary Table S3.

Supplementary Table S4.

## Data Availability

The DNA-seq data generated and analysed during the current study have been deposited at the NCBI Sequence Read Archive with the Bioproject ID: PRJNA560333.

## Abbreviations

GS/MS: Gas chromatograph-mass spectrometer
PCoA: Principal co-ordinates analysis
NMDS: Nonmetric multidimensional scaling
Cys-Gly: Cysteinylglycine
TICs: Total ion currents

## References

[1] Xiong W (2015). Different continuous cropping spans significantly affect microbial community membership and structure in a vanilla-grown soil as revealed by deep pyrosequencing. Microb. Ecol..

[2] Li X (2016). Effects of long-term continuous cropping on soil nematode community and soil condition associated with replant problem in strawberry habitat. Sci. Rep..

[3] Tan Y (2017). Diversity and composition of rhizospheric soil and root endogenous bacteria in *Panax notoginseng* during continuous cropping practices. J. Basic Microbiol..

[4] Wang C (2007). Effect of continuous cropping on photosynthesis and metabolism of reactive oxygen in peanut. Agron. Crop Ecol..

[5] Zhu S-Y, Liu T-M, Tang Q-M, Tang S-W (2014). Influence of different continuous cropping obstacle factor on agronomic traits of ramie. Plant Fiber Sciences in China.

[6] Akinrotimi C, Okocha P (2018). Effects of planting year and genotypes on the seed yield of Kenaf (*Hibiscus cannabinus* L.). J. Plant Sci. Agric. Res..

[7] Tan Y (2017). Rhizospheric soil and root endogenous fungal diversity and composition in response to continuous *Panax notoginseng* cropping practices. Microbiol. Res..

[8] Xiong W (2015). The effect of long-term continuous cropping of black pepper on soil bacterial communities as determined by 454 pyrosequencing. PLoS ONE.

[9] Qin S (2017). Breaking continuous potato cropping with legumes improves soil microbial communities, enzyme activities and tuber yield. PLoS ONE.

[10] Kagale S (2016). The developmental transcriptome atlas of the biofuel crop *Camelina sativa*. Plant J..

[11] Han J, Zhang  J-w, Xu W-x, Luo M, Wu L-l (2011). Dynamics analysis of culturable soil microflora and microbial activity in continuous and rotation cropping systems of Xinjiang cotton. Cotton Sci..

[12] Zhu S (2018). Potential use of high-throughput sequencing of soil microbial communities for estimating the adverse effects of continuous cropping on ramie (*Boehmeria nivea* L. Gaud). PLoS ONE.

[13] Amalfitano C (2018). Plant-rhizobium symbiosis, seed nutraceuticals, and waste quality for energy production of *Vicia faba* L. as affected by crop management. Chem. Biol. Technol. Agric..

[14] da Silva Júnior EB, Favero VO, Xavier GR, Boddey RM, Zilli JE (2018). Rhizobium inoculation of cowpea in Brazilian cerrado increases yields and nitrogen fixation. Agron. J..

[15] Nyoki D, Ndakidemi PA (2018). Selected chemical properties of soybean rhizosphere soil as influenced by cropping systems, rhizobium inoculation, and the supply of phosphorus and potassium after two consecutive cropping seasons. Int. J. Agron..

[16] Li J, Chen X, Zhan R, He R (2019). Transcriptome profiling reveals metabolic alteration in *Andrographis paniculata* in response to continuous cropping. Ind. Crops Prod..

[17] Li Z (2016). High-efficiency ramie fiber degumming and self-powered degumming wastewater treatment using triboelectric nanogenerator. Nano Energy.

[18] Kundu P, Sarkar C (1996). Chemical degumming and fibre characteristics of ramie at different stages of crop growth. Indian J. Fibre Text. Res..

[19] Ramesh M (2016). Kenaf (*Hibiscus cannabinus* L.) fibre based bio-materials: a review on processing and properties. Progr. Mater. Sci..

[20] Fu Q (2012). Soil microbial communities and enzyme activities in a reclaimed coastal soil chronosequence under rice–barley cropping. J. Soils Sediments.

[21] Bai L, Cui J, Jie W, Cai B (2015). Analysis of the community compositions of rhizosphere fungi in soybeans continuous cropping fields. Microbiol. Res..

[22] Zhao J (2020). Dissecting the effect of continuous cropping of potato on soil bacterial communities as revealed by high-throughput sequencing. PLoS ONE.

[23] She S (2017). Significant relationship between soil bacterial community structure and incidence of bacterial wilt disease under continuous cropping system. Arch. Microbiol..

[24] Li YC (2016). Variations of rhizosphere bacterial communities in tea (*Camellia sinensis* L.) continuous cropping soil by high-throughput pyrosequencing approach. J. Appl. Microbiol..

[25] Qin XM, Zheng Y, Tang L, Long GQ (2017). Crop rhizospheric microbial community structure and functional diversity as affected by maize and potato intercropping. J. Plant Nutr..

[26] Marion, C., Sall, S., Chotte, J.-L. & Lesueur, D. Soil microbial functionning affected by rhizobial inoculation and arabic gum production in mature *Acacia Senegal* plantation. Soil microbial functionning affected by rhizobial inoculation and arabic gum production in mature *Acacia senegal* plantation. In:*11th Congress of African Association for Biological Nitrogen Fixation: Impact of biological nitrogen fixation on agricultural development in Africa (Abstracts)*, November 22-27, (2004).

[27] Sun YM (2009). Influence of intercropping and intercropping plus rhizobial inoculation on microbial activity and community composition in rhizosphere of alfalfa (*Medicago sativa* L.) and Siberian wild rye (*Elymus sibiricus* L.). FEMS Microbiol. Ecol..

[28] Teng Y (2011). Influence of *Rhizobium meliloti* on phytoremediation of polycyclic aromatic hydrocarbons by alfalfa in an aged contaminated soil. J. Hazard Mater..

[29] Bakhoum N (2012). Impact of rhizobial inoculation on *Acacia senegal* (L.) Willd. growth in greenhouse and soil functioning in relation to seed provenance and soil origin. World J. Microbiol. Biotechnol..

[30] Guarino C (2019). Investigation and assessment for an effective approach to the reclamation of polycyclic aromatic hydrocarbon (PAHs) contaminated site: SIN Bagnoli, Italy. Sci. Rep..

[31] Mohite B (2013). Isolation and characterization of indole acetic acid (IAA) producing bacteria from rhizospheric soil and its effect on plant growth. J. Soil Sci. Plant Nutr..

[32] Zhao Y (2010). Auxin biosynthesis and its role in plant development. Annu. Rev. Plant Biol..

[33] Cohen AC (2015). Azospirillum brasilense ameliorates the response of *Arabidopsis thaliana* to drought mainly via enhancement of ABA levels. Physiol. Plant..

[34] Amavizca E (2017). Enhanced performance of the microalga *Chlorella sorokiniana* remotely induced by the plant growth-promoting bacteria *Azospirillum brasilense* and *Bacillus pumilus*. Sci. Rep..

[35] Crozier A, Arruda P, Jasmim JM, Monteiro AM, Sandberg G (1988). Analysis of indole-3-acetic acid and related indoles in culture medium from *Azospirillum lipoferum* and *Azospirillum brasilense*. Appl. Environ. Microbiol..

[36] Pii Y, Aldrighetti A, Valentinuzzi F, Mimmo T, Cesco S (2019). *Azospirillum brasilense* inoculation counteracts the induction of nitrate uptake in maize plants. J. Exp. Bot..

[37] Santos K (2017). Wheat colonization by an *Azospirillum brasilense* ammonium-excreting strain reveals upregulation of nitrogenase and superior plant growth promotion. Plant Soil.

[38] Park S-I (2017). Improved stress tolerance and productivity in transgenic rice plants constitutively expressing the *Oryza sativa* glutathione synthetase OsGS under paddy field conditions. J. Plant Physiol..

[39] Capaldi FR, Gratão PL, Reis AR, Lima LW, Azevedo RA (2015). Sulfur metabolism and stress defense responses in plants. Trop. Plant Biol..

[40] Bohnert HJ, Jensen RG (1996). Strategies for engineering water-stress tolerance in plants. Trends Biotechnol..

[41] Zhao Y (2016). Heterocyclic aromatic N-oxidation in the biosynthesis of phenazine antibiotics from *Lysobacter antibioticus*. Org. Lett..

[42] Ko HS, Jin RD, Krishnan HB, Lee SB, Kim KY (2009). Biocontrol ability of *Lysobacter antibioticus* HS124 against Phytophthora blight is mediated by the production of 4-hydroxyphenylacetic acid and several lytic enzymes. Curr. Microbiol..

[43] Purcell LCS, Ashlock L (2013). Arkansas Soybean Handbook.

[44] Wani PA, Khan MS, Zaidi A (2007). Effect of metal tolerant plant growth promoting *Bradyrhizobium* sp. (vigna) on growth, symbiosis, seed yield and metal uptake by greengram plants. Chemosphere.

[45] Antoun H, Beauchamp CJ, Goussard N, Chabot R, Lalande R (1998). Molecular Microbial Ecology of the Soil.

[46] Egamberdieva D, Reckling M, Wirth S (2017). Biochar-based Bradyrhizobium inoculum improves growth of lupin (*Lupinus angustifoliu*s L.) under drought stress. Eur. J. Soil Biol..

[47] Laborda P, Zhao Y, Ling J, Hou R, Liu F (2018). Production of antifungal p-aminobenzoic acid in *Lysobacter antibioticus* OH13. J. Agric. Food Chem..

[48] Fu L (2018). Antifungal and biocontrol evaluation of four lysobacter strains against clubroot disease. Indian J. Microbiol..

[49] Liu M (2017). Improved nutrient status affects soil microbial biomass, respiration, and functional diversity in a Lei bamboo plantation under intensive management. J. Soils Sediments.

[50] Ullah S (2017). Interactive effect of gibberellic acid and NPK fertilizer combinations on ramie yield and bast fibre quality. Sci. Rep..

[51] Luo R (2012). SOAPdenovo2: an empirically improved memory-efficient short-read de novo assembler. Gigascience.

[52] Li W, Jaroszewski L, Godzik A (2001). Clustering of highly homologous sequences to reduce the size of large protein databases. Bioinformatics.

[53] Langmead B, Salzberg SL (2012). Fast gapped-read alignment with Bowtie 2. Nat. Methods.

