# Supplementary material for: Identification of the rhizospheric microbe and metabolites that led by the continuous cropping of ramie (Boehmeria nivea L. Gaud)
Source: Sci Rep. 2020 Nov 23;10:20408. doi: 10.1038/s41598-020-77475-3 (PMC7683709; doi:10.1038/s41598-020-77475-3)
Supplement: Supplementary file 1 — Supplementary Legends. [file 41598_2020_77475_MOESM1_ESM.docx]

**Supplementary files:** Table S1. The summary of the Illumina sequencing; Figure S1. The heatmap of the different bacterial family between the two groups. Health-group, soil without continuous cropping; Table S2. Microbe abundance in all samples; Figure S2. The PCA (A) and OPLS-DA (B) of the soil and root samples, (C) showed the validation results of OPLS-DA; Table S3. The differential metabolites in soil and root samples between Health-group (without obstacle) and Obstacle-group (with obstacle); Table S4. The top 20 KEGG pathways associated with the 31 differential metabolites in root samples.
