# Supplementary figures and images for: Identification of the rhizospheric microbe and metabolites that led by the continuous cropping of ramie (Boehmeria nivea L. Gaud)
Source: Sci Rep. 2020 Nov 23;10:20408. doi: 10.1038/s41598-020-77475-3 (PMC7683709; doi:10.1038/s41598-020-77475-3)

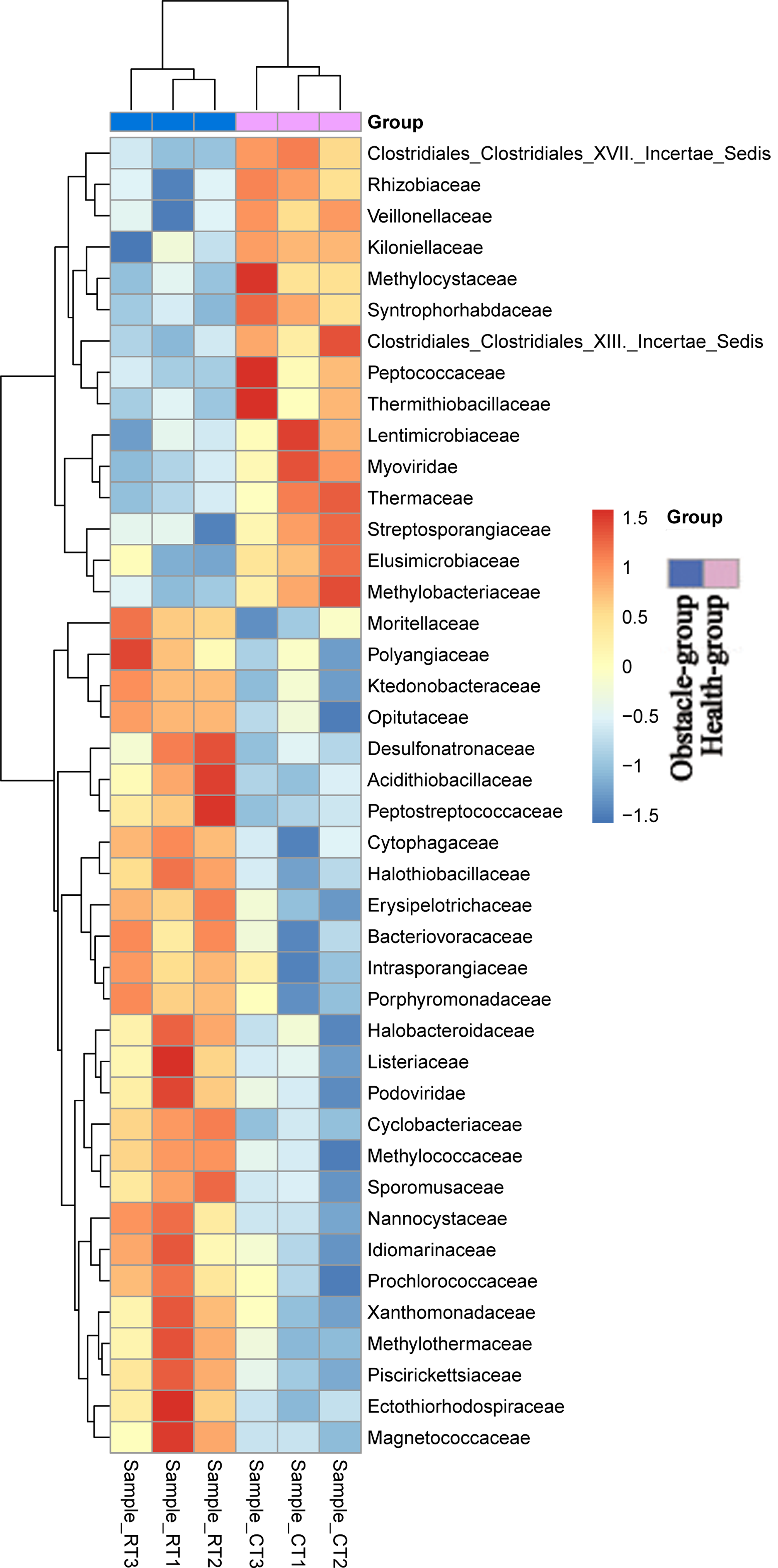

Supplement: Supplementary file 2 — Supplementary Figure S1. [file 41598_2020_77475_MOESM2_ESM.tif]

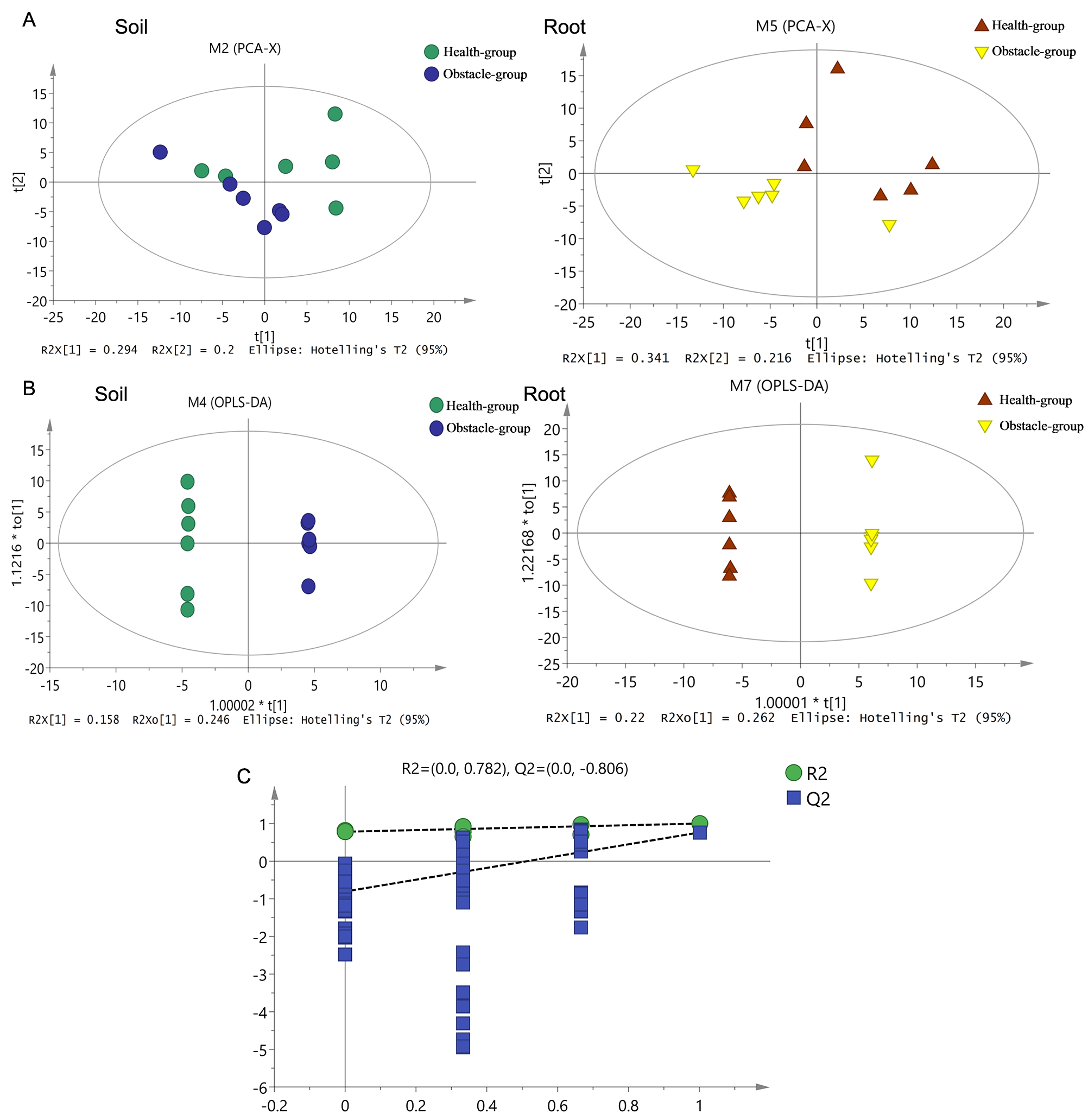

Supplement: Supplementary file 3 — Supplementary Figure S2. [file 41598_2020_77475_MOESM3_ESM.tif]
