# Supplementary material for: Identification of the rhizospheric microbe and metabolites that led by the continuous cropping of ramie (Boehmeria nivea L. Gaud)
Source: Sci Rep. 2020 Nov 23;10:20408. doi: 10.1038/s41598-020-77475-3 (PMC7683709; doi:10.1038/s41598-020-77475-3)
Supplement: Supplementary file 4 — Supplementary Table S1. [file 41598_2020_77475_MOESM4_ESM.docx]

Identification of the rhizospheric microbe and metabolites that led by the continuous cropping of ramie (*Boehmeria nivea* L. Gaud)

Yanzhou Wang^1^, Siyuan Zhu^1,^ *, Touming Liu^1^, Bing Guo^1^, Fu Li^1^ and Xuehua Bai^1^

^1^Institute of bast fiber crops, Chinese Academy of Agricultural Sciences, Changsha Hunan 410205, P.R. China.

Corresponding authors:

* Corresponding author: Pro.Siyuan Zhu

Email: [zhusiyuan@caas.cn](mailto:zhusiyuan@caas.cn)

Co-authors:

Yanzhou Wang: [wyzhcf@163.com](mailto:wyzhcf@163.com)

Touming Liu: [liutouming@gmail.com](mailto:liutouming@gmail.com)

Bing Guo: [guobing@caas.cn](mailto:guobing@caas.cn)

Fu Li: lifu7045@qq.com

Xuehua Bai: 18235707017@163.com

Table S1. The summary of the Illumina sequencing.

| Group | Sample ID | Reads | Base | >Q30% | GC% | Scaffold | ORFs count | Bacterial reads (%) |
| --- | --- | --- | --- | --- | --- | --- | --- | --- |
| A | CT1 | 56130711 | 8.4E+09 | 94.31 | 64.83 | 1941026 | 91393 | 98.27 |
|  | CT2 | 54816094 | 8.14E+09 | 94.56 | 64.78 | 1765185 | 87720 | 98.30 |
|  | CT3 | 62870946 | 9.34E+09 | 94.69 | 64.61 | 1952944 | 104309 | 98.25 |
| B | RT1 | 57687759 | 8.57E+09 | 94.77 | 65.06 | 1573610 | 64036 | 98.25 |
|  | RT2 | 60857059 | 9.1E+09 | 94.61 | 64.48 | 1894792 | 84400 | 98.23 |
|  | RT3 | 50082086 | 7.43E+09 | 94.57 | 64.41 | 1322867 | 48973 | 98.26 |
|  |  | 326.58 M | 47.48 G | 94.59 | 64.37 | 10450424 | 480931 | 98.26 |
